# CARD9 contributes to ovarian cancer cell proliferation, cycle arrest, and cisplatin sensitivity

**DOI:** 10.1186/s12860-022-00447-0

**Published:** 2022-11-28

**Authors:** Yanming Wang, Chao Wang, Yan Zhu

**Affiliations:** 1 Department of Obstetrics and Gynecology, the first Affiliated Hospital of Jinzhou Medical University, No. 2, Section 5, Renmin Street, Jinzhou, 121000 Liaoning China; 2 Department of Otolaryngology, the 968th Hospital of the PLA Joint Logistic Support Force, No. 9, Section 2, Chongqing Road, Jinzhou, 121000 Liaoning China

**Keywords:** CARD9, HOXB5, Ovarian cancer, Cycle arrest, Cisplatin sensitivity

## Abstract

**Background:**

Ovarian cancer recurrence and chemotherapy resistance are still urgent issues, and exploring the mechanisms of metastasis and chemotherapy resistance is beneficial to the development of therapeutic methods. Caspase recruitment domain family member 9 (CARD9) and homeobox B5 (HOXB5) are related and both are upregulated in ovarian cancer. This study aimed to define their functions in ovarian cancer cell proliferation, migration, and cisplatin sensitivity.

**Results:**

The levels of CARD9 were detected in acquired ovarian cancer tissues and cell lines. CARD9 was indeed abnormally upregulated in them. CARD9 knockdown significantly suppressed cell proliferation, colony formation, migration, cycle arrest, and cisplatin sensitivity. HOXB5 bound to the CARD9 promoter, and HOXB5 overexpression reversed the regulation by CARD9 knockdown in cells, as well as the activation of NF-κB signaling. This indicated that CARD9 was positively regulated by HOXB5 in ovarian cancer cells.

**Conclusion:**

Together, CARD9 is involved in ovarian cancer cell proliferation, migration, and cisplatin sensitivity via NF-κB signaling after transcriptional activation by HOXB5.

**Supplementary Information:**

The online version contains supplementary material available at 10.1186/s12860-022-00447-0.

## Introduction

Ovarian cancer is a type of lethal gynecological malignancy in women, and its general characteristics are ambiguous early clinical symptoms, late diagnosis, and a dismal survival rate [1]. Overall 5-year survival is currently approaching 90% in the early stages (I + II), but just 29% in the advanced stages (III + IV) [2]. Tumor debulking surgery and platinum/taxane chemotherapy are currently the standards of care for ovarian cancer, however, most patients relapse and develop chemotherapy resistance, with a five-year survival rate of < 35% [3]. Therefore, there is an urgent need to investigate the molecular mechanisms of ovarian cancer metastasis and chemotherapy resistance, as well as to identify effective prognostic biomarkers to ameliorate the development of ovarian cancer.

Caspase recruitment domain family member 9 (CARD9) is a member of the CARD protease family that is distributed in various tissues throughout the human body and represents an essential regulatory role in the activation of caspase and NF-κB in inflammation and immunity [4]. A study discovered that CARD9 was a crucial regulator of tumor formation [5] and a promising therapeutic target for lung cancer [6]. Through the NF-κB/indoleamine 2, 3-dioxygenase pathway, it can prevent the development of lung cancer [7]. Downregulation of CARD9 suppressed the growth of oral squamous cell carcinoma by controlling NF-κB [8], and also prevented esophageal squamous cell carcinoma proliferation and migration [9]. Furthermore, according to research, RAD50 double strand break repair protein-mediated NF-κB pathway activation in ovarian cancer is dependent on CARD9, and interfering with CARD9 reduces ovarian cancer cell migration [10]. Herein, CARD9 expression was found to be up-regulated in ovarian cancer using the Gene Expression Profiling Interactive Analysis (GEPIA) database analysis (gepia.cancer-pku.cn) [11], and high expression was associated with a poor prognosis. As a result, CARD9 is proposed to be associated with the onset and progression of ovarian cancer.

The interaction between CARD9 and homeobox B5 (HOXB5) was found using the Biological General Repository for Interaction Datasets (BioGRID) database (thebiogrid.org) [12]. HOXB5 is a member of the homeobox gene family and has been found to be involved in the development of various cancers [13–15]. According to the JASPAR database (jaspar.genereg.net) [16], there is a binding site for the transcription factor HOXB5 in the promoter region of CARD9. Hence, we hypothesized that HOXB5 regulated CARD9 transcription. The major objective of this work was to define the functions of CARD9 and HOXB5 in ovarian cancer cell proliferation, migration, and cisplatin sensitivity.

## Results

### CARD9 levels in ovarian cancer tissues and cells

**Fig. 1:**
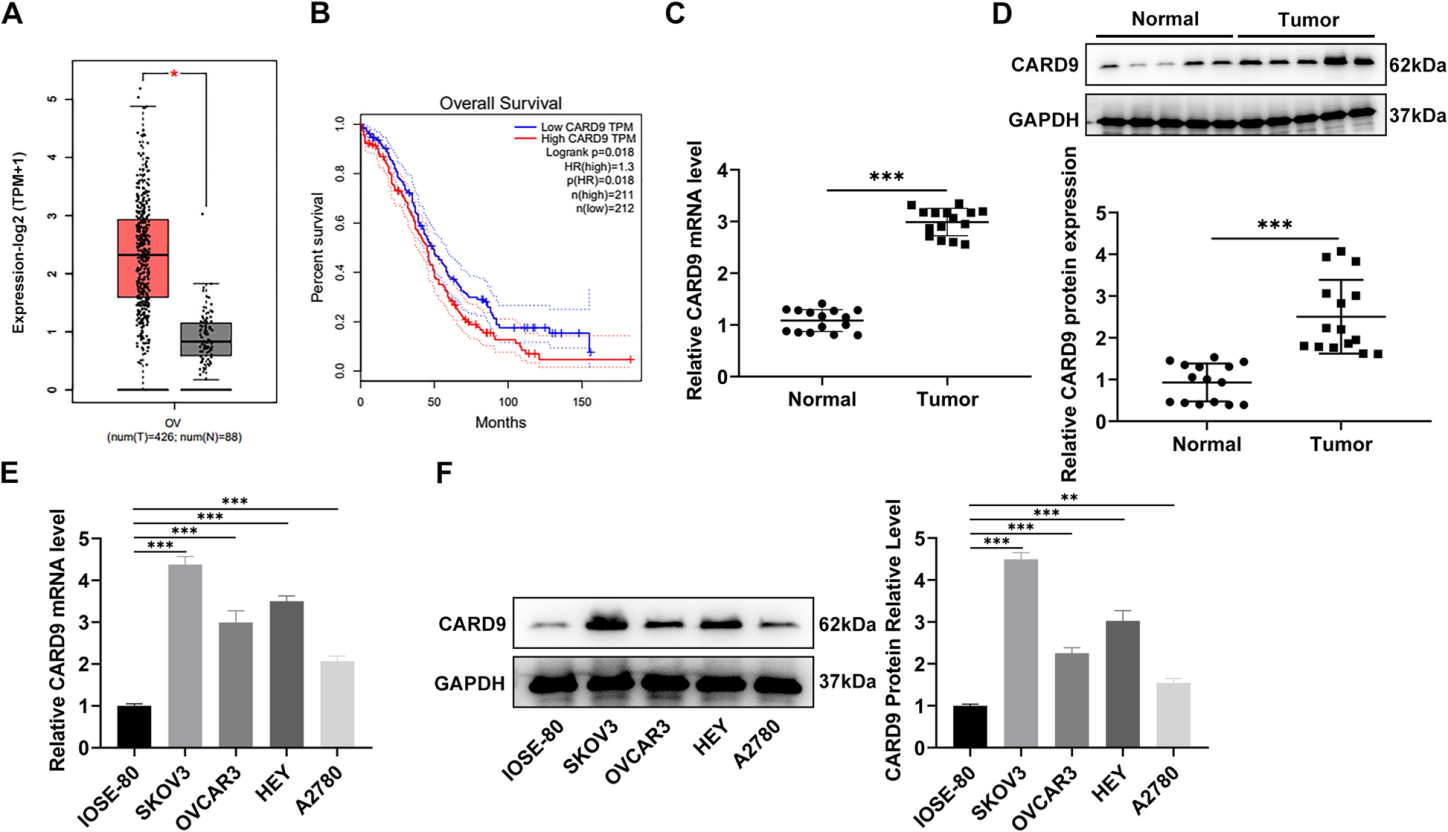
CARD9 levels in ovarian cancer tissues and cells. **A** According to the GEPIA database, the level of CARD9 in ovarian cancer was analyzed. **B** A high level of CARD9 is positively correlated with poor prognosis. **C** The level of CARD9 in collected ovarian cancer and adjacent tissue samples was detected by RT-qPCR and (**D**) immunoblotting. **E** The levels of CARD9 in ovarian cancer cell lines were detected by RT-qPCR and (**F**) immunoblotting. ^**^P < 0.01, ^***^P < 0.001 vs. IOSE-80

According to the GEPIA database, the level of CARD9 is significantly elevated in ovarian cancer (Fig. 1A) and its high level is positively correlated with poor prognosis (Fig. 1B). So we actually collected ovarian cancer and adjacent tissue samples, and analyzed the level of CARD9 by RT-qPCR and immunoblotting, respectively. From the presented results, it was shown that CARD9 was indeed abnormally upregulated in ovarian cancer tissue (Fig. 1C, D). CARD9 levels in the cell lines were then assessed, and the results were consistent with trends observed in clinical samples (Fig. 1E, F).

### Effects of CARD9 on proliferation and migration

**Fig. 2:**
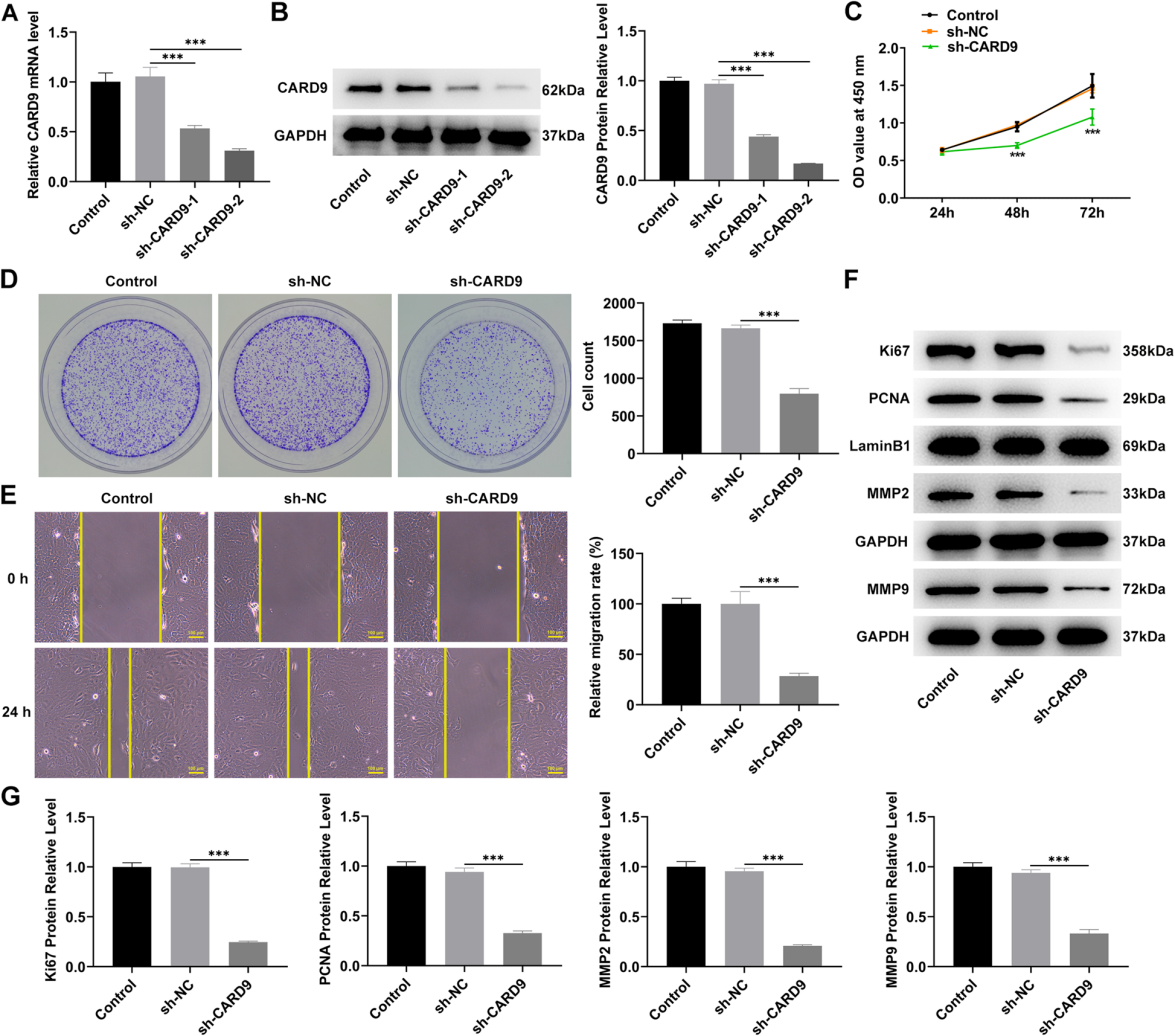
Effects of CARD9 on proliferation and migration. **A** The level of CARD9 in the transfected SKOV3 cell line was detected by RT-qPCR and (**B**) immunoblotting. **C** The effects of CARD9 knockdown on cell proliferation, (**D**) colony formation, and (**E**) migration were evaluated using the CCK8, colony formation, and wound healing assays. **F**, **G** The levels of proliferation- and migration-related proteins were determined using immunoblotting. ^***^P < 0.001 vs. sh-NC

To explore the specific roles of CARD9, the CARD9 level in cells was knocked down, and the results displayed that CARD9 in the transfected SKOV3 cell line could be significantly reduced (Fig. 2A, B). The group with a more pronounced CARD9 knockdown, sh-CARD9-2, was selected for cell proliferation experiments. The CCK-8 results revealed that the proliferation of cells with CARD9 knockdown was significantly reduced (Fig. 2C). In addition, the ability of colony formation (Fig. 2D) and migration (Fig. 2E) was severely impaired after the CARD9 knockdown. Meanwhile, the levels of proliferation- and migration-related proteins were determined (Fig. 2F, G). From the results of immunoblotting, CARD9 knockdown triggered a decrease in the levels of Ki67, proliferating cell nuclear antigen (PCNA), matrix metallopeptidase (MMP)2, and MMP9 proteins.

### Effects of CARD9 on cycle arrest and cisplatin sensitivity

**Fig. 3:**
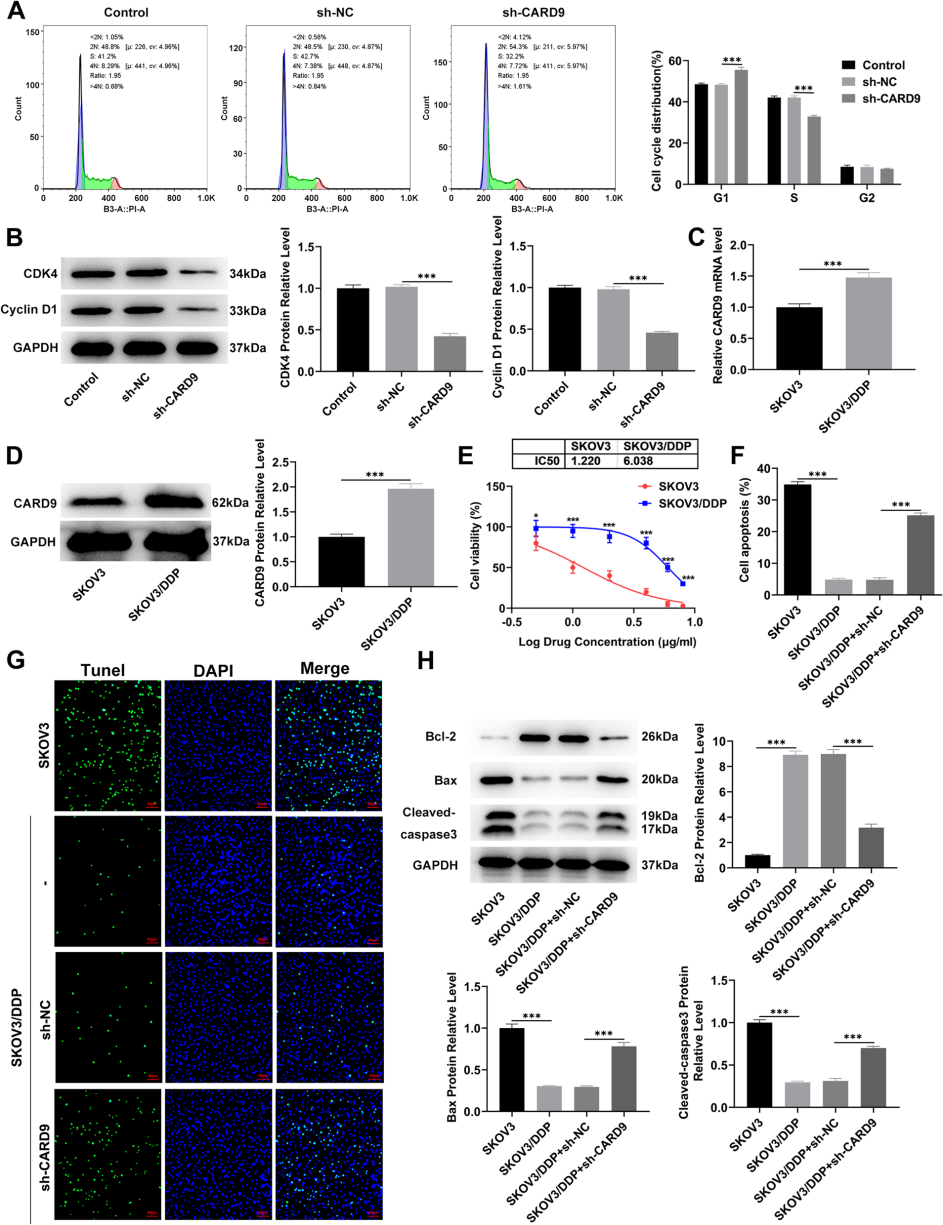
Effects of CARD9 on cycle arrest and cisplatin sensitivity. **A** Cell cycle distribution was assessed by flow cytometry. **B** The levels of cycle-related proteins in cells were detected by immunoblotting. **C** CARD9 levels in SKOV3/DDP and SKOV3 cell lines were determined using RT-qPCR and (**D**) immunoblotting. **E** The effect of different concentrations of DDP on the viability of SKOV3 and SKOV3/DDP cell lines was determined using the CCK8 assay. **F**, **G** The level of apoptosis was assessed by TUNEL staining and (H) immunoblotting. ^*^P < 0.05, ^***^P < 0.001 vs. sh-NC or SKOV3; ^###^*P* < 0.001 vs. SKOV3/DDP + sh-NC

Cell cycle distribution was assessed by flow cytometry. Compared with control, more cells were arrested in the G1 phase, indicating that CARD9 knockdown could cause cell cycle arrest (Fig. 3A). The levels of cycle-related proteins in cells were detected by immunoblotting. The levels of CDK4 and cyclin D1 were significantly decreased in cells with CARD9 knockdown (Fig. 3B). Afterward, CARD9 levels in SKOV3/ Cisplatin (cis-dichlorodiammine platinum, DDP) cell line were compared with those in the SKOV3 cell line. The results indicated higher levels of CARD9 in the DDP-resistant cell lines (Fig. 3C, D). In addition, the Cell Counting Kit-8 (CCK8) assay was used to evaluate the effect of different concentrations of DDP on the viability of SKOV3 and SKOV3/DDP cell lines. SKOV3 cell line was more sensitive to DDP exposure and had a obviously lower IC_50_ value than the SKOV3/DDP cell line (Fig. 3E). Furthermore, after 1 μg/ml DDP treatment, the level of apoptosis was assessed by TUNEL staining (Fig. 3F, G) and immunoblotting (Fig. 3H). According to the fluorescence intensity, the apoptosis in the SKOV3/DDP group was significantly less as compared to in the SKOV3 group, whereas the apoptosis level in SKOV3/DDP cell line with CARD9 knockdown was significantly higher than that in the untransfected SKOV3/DDP cell line. CARD9 knockdown significantly reduced Bcl-2 levels in SKOV3/DDP cells, and was accompanied by increased levels of Bax and cleaved-caspase3 compared to untransfected SKOV3/DDP cells.

### Association between HOXB5 and CARD9

**Fig. 4:**
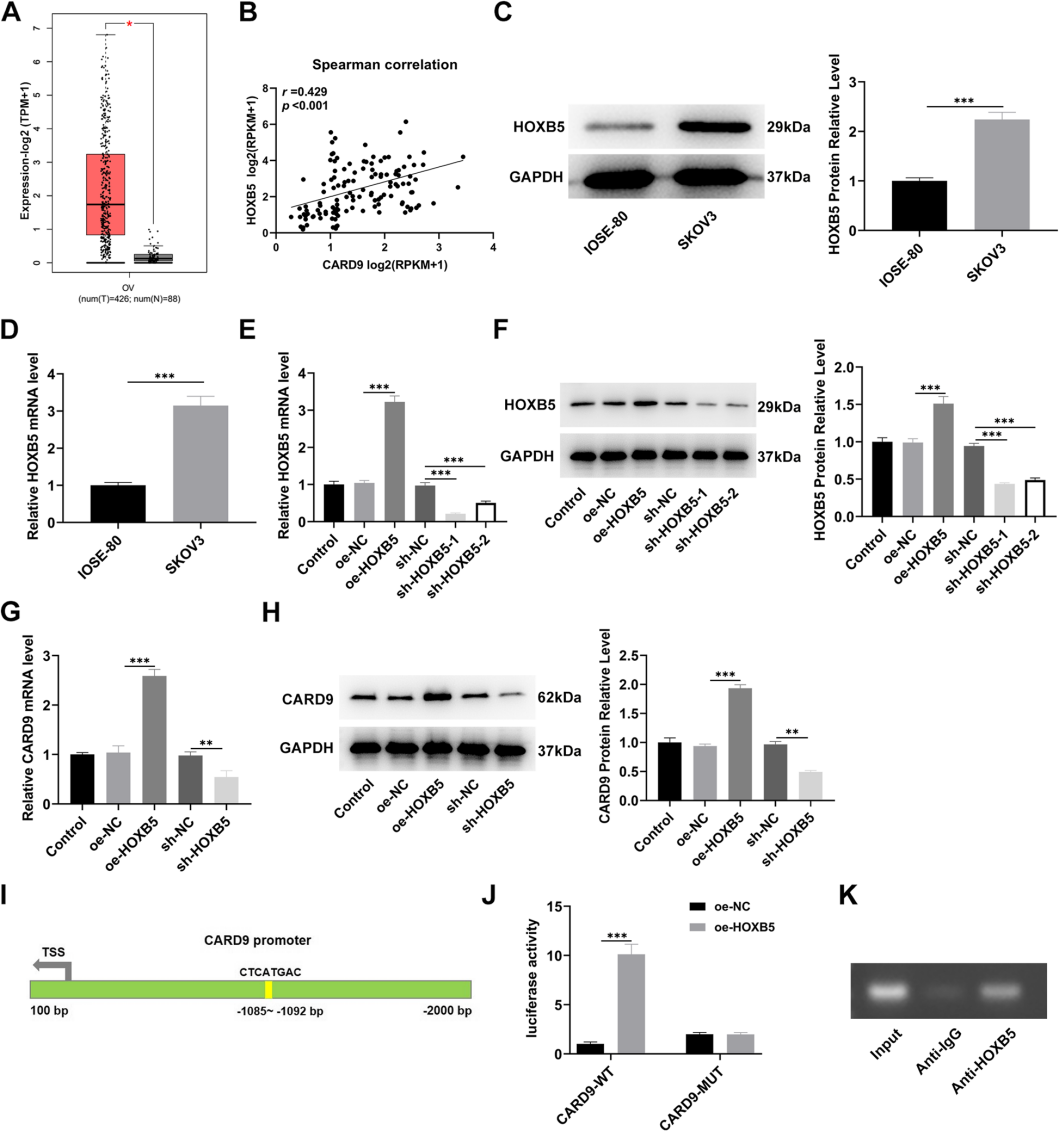
Association between HOXB5 and CARD9. **A** According to the GEPIA database, the level of HOXB5 in ovarian cancer was analyzed. **B** HOXB5 is positively correlated with CARD9 according to Spearman correlation. **C** The level of HOXB5 in ovarian cancer cell lines was detected by immunoblotting and (**D**) RT-qPCR. **E** The efficacy of HOXB5 overexpression and knockdown in SKOV3 cells were assessed by RT-qPCR and (**F**) immunoblotting. **G** HOXB5 overexpression could lead to increased levels of CARD9 from RT-qPCR and (**H**) immunoblotting results. **I** According to the JASPAR database, there is a binding site for the transcription factor HOXB5 in the promoter region of CARD9. **J** The association between CARD9 and HOXB5 was verified using dual-luciferase reporter assay and (K) ChIP. ^***^P < 0.001 vs. oe-NC or IOSE-80; ^##^*P* < 0.01, ^###^*P* < 0.001 vs. sh-NC

GEPIA analysis demonstrated that HOXB5 was also highly expressed in ovarian cancer tissues (Fig. 4A) and positively correlated with CARD9 according to Spearman correlation (Fig. 4B). The upward trend of HOXB5 in tissues was consistent with the results in cells (Fig. 4C, D). After confirming that HOXB5 overexpression and knockdown were successfully achieved in SKOV3 cells (Fig. 4E, F), CARD9 levels in these transfected cells were examined. RT-qPCR and immunoblotting results revealed that HOXB5 overexpression could lead to increased levels of CARD9, and vice versa (Fig. 4G, H). This suggested that HOXB5 and CARD9 might be in a regulatory axis. Based on the predicted binding location (Fig. 4I), a dual-luciferase reporter assay was then used to assess the effect of site mutation on the promoter region (Fig. 4J). Upon mutation, luciferase activity was significantly decreased. Then chromatin immunoprecipitation (ChIP) -PCR was also used to confirm the binding of HOXB5 to the CARD9 promoter (Fig. 4K). The enrichment of the harvested CARD9 was markedly higher than the IgG group.

### Effects of HOXB5 on CARD9 regulation and NF-κB signaling

**Fig. 5:**
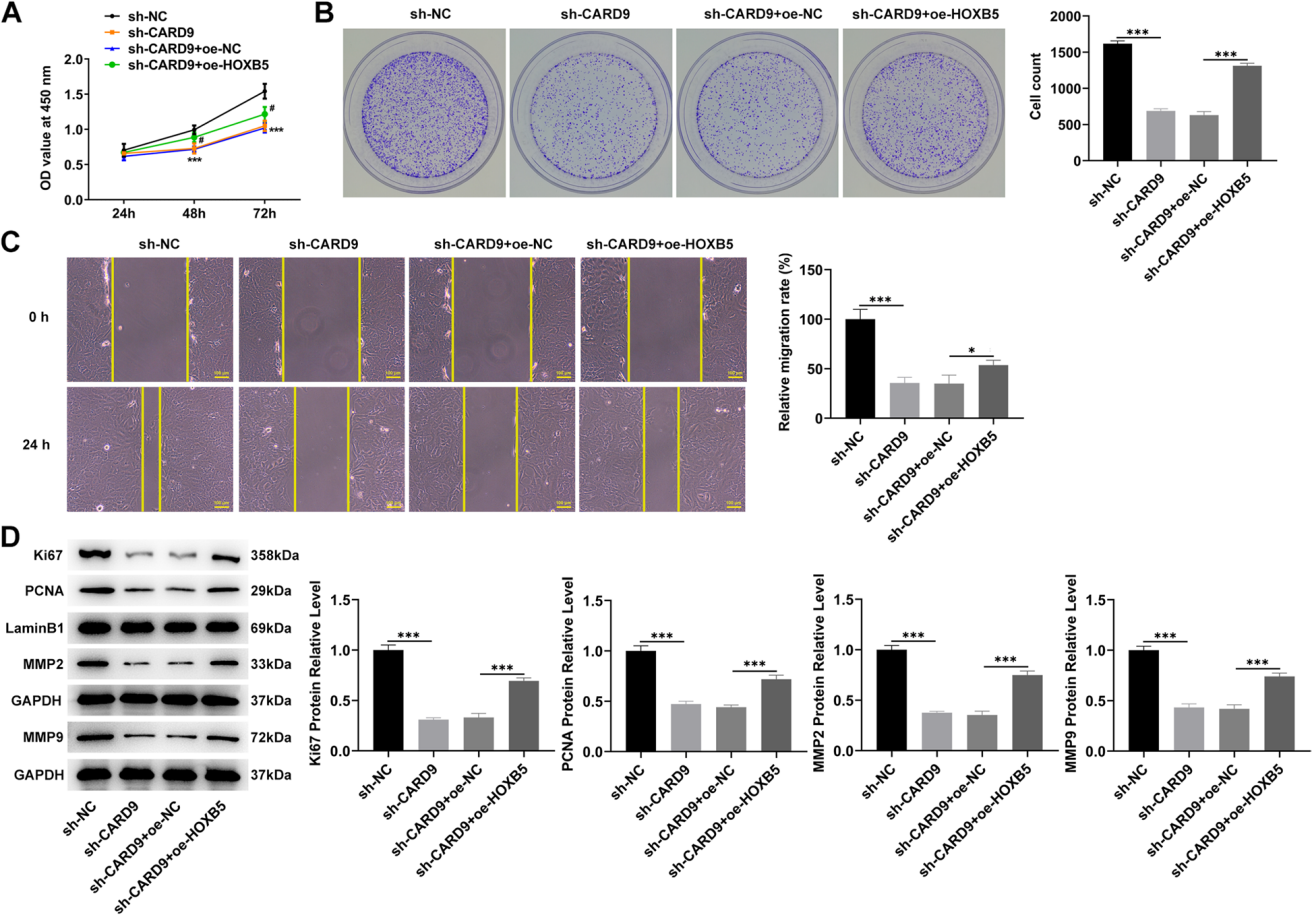
Effects of HOXB5 on CARD9 regulation. **A** The effects of HOXB5 overexpression on cell proliferation, (**B**) colony formation, and (**C**) migration were evaluated. **D** The levels of proliferation- and migration-related proteins were determined using immunoblotting. ^***^P < 0.001 vs. sh-NC; ^#^*P* < 0.05, ^###^*P* < 0.001 vs. sh-CARD9 + oe-NC

**Fig. 6:**
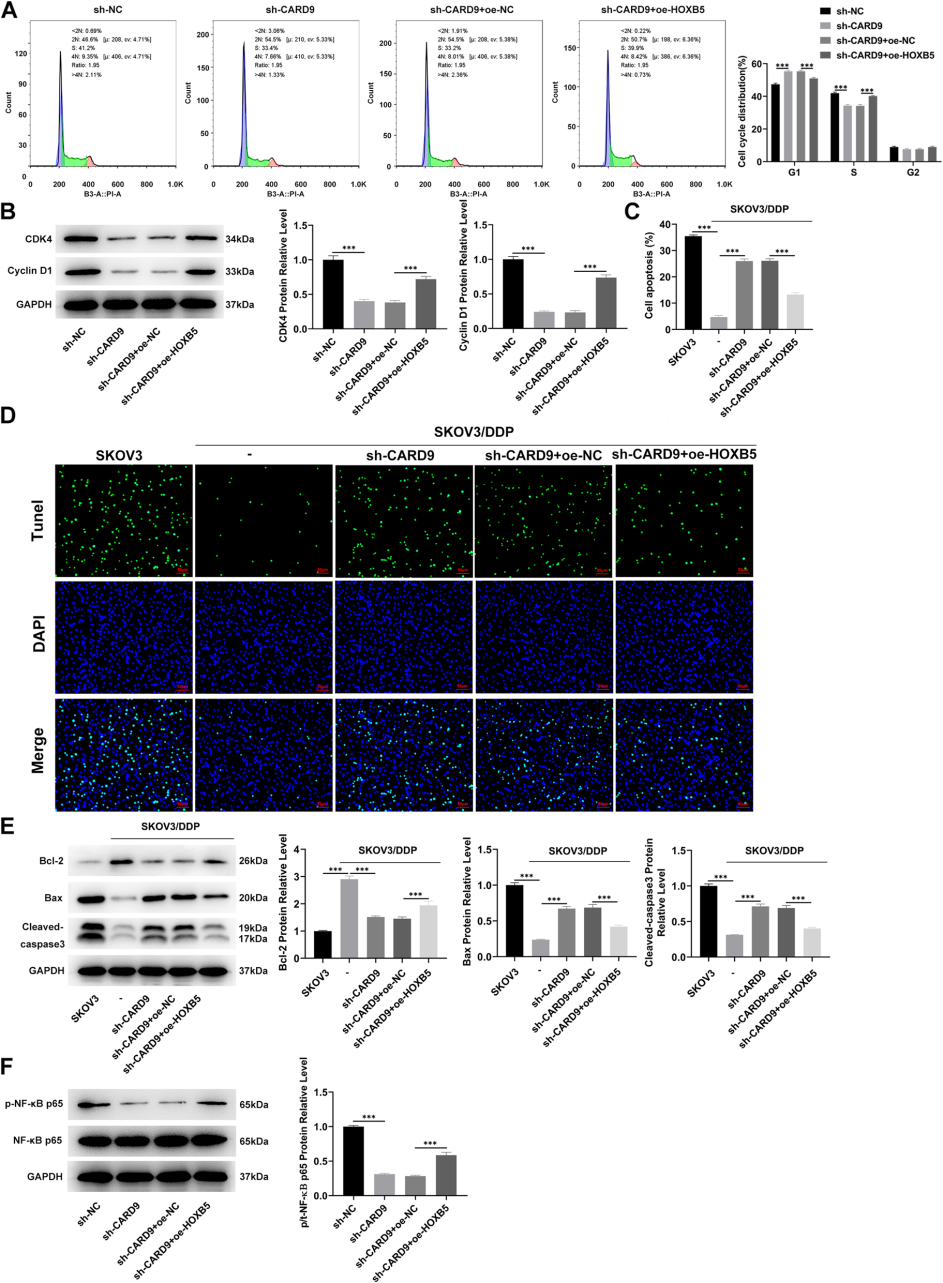
Effects of HOXB5 on CARD9 regulation and NF-κB signaling. **A** Cell cycle distribution was assessed by flow cytometry. **B** The levels of cycle-related proteins in cells were detected by immunoblotting. **C**, **D** The level of apoptosis was assessed by TUNEL staining and (**E**) immunoblotting. **F** The levels of NF-κB signaling pathway-related proteins in cells were detected by immunoblotting. ^***^P < 0.001 vs. sh-NC; ^#^*P* < 0.05, ^###^*P* < 0.001 vs. SKOV3/DDP; ^@@@^*P* < 0.001 vs. SKOV3/DDP + sh-CARD9 + oe-NC

The effects of HOXB5 overexpression on cell proliferation (Fig. 5A), colony formation (Fig. 5B), migration (Fig. 5C), and related protein levels (Fig. 5D) were subsequently evaluated. Compared with the CARD9 knockdown group, the additional increase in the HOXB5 level caused the promotion of the above-mentioned aspects, partially reversing the effect of CARD9 knockdown on cells. Moreover, the effect of HOXB5 overexpression on the cell cycle and cisplatin sensitivity was also evaluated. HOXB5 overexpression could alleviate the cell cycle arrest caused by CARD9 knockdown and promote cycle progression (Fig. 6A, B). Apoptosis in SKOV3/DDP cells with additional HOXB5 overexpression could alleviate the cell apoptosis caused by CARD9 knockdown (Fig. 6C, E). Finally, the levels of NF-κB signaling pathway-related proteins in cells were detected by immunoblotting (Fig. 6F). CARD9 knockdown resulted in a decrease in the level of phosphorylated NF-κB p65 protein, which was increased by HOXB5 overexpression. This indicated that CARD9 and HOXB5 could promote the activation of NF-κB p65 signaling.

## Discussion

Surgery and postoperative platinum and paclitaxel chemotherapy have always been the preferred chemotherapy regimens recommended by the National Comprehensive Cancer Network for ovarian cancer [17]. However, approximately 60% of ovarian cancer patients who receive chemotherapy will experience tumor progression or recurrence, with nearly half experiencing their first recurrence within a year of discontinuing first-line platinum-based therapy, and nearly a quarter experiencing recurrence within 6 months [18, 19]. Response rates to routinely used cytotoxic drugs in platinum-resistant patients are < 20%, and randomized phase III clinical trials have indicated progression-free survival of 3-4 months and median overall survival of less than 1 year [20]. Despite significant advances in combined chemotherapy and targeted therapy in recent years, the overall progression-free survival of platinum-resistant patients has the least impact, and statistics show that the proportion of such patients is on the rise. Platinum resistance in advanced ovarian cancer patients climbed to 70-80% in 2019 [21]. Therefore, we explore the roles of CARD9 and HOXB5 in ovarian cancer cells in relation to cisplatin sensitivity. These results suggest that CARD9 is involved in cisplatin resistance in ovarian cancer cells after transcriptional activation by HOXB5.

Previous studies have found that HOXB5 is up-regulated in breast cancer and can enhance tumor growth via the Wnt/β-catenin pathway [22], and its transcriptional activation of EGFR promotes tumor cell invasion [23]. Moreover, HOXB5 is up-regulated in tumors such as endometrial [24], liver [25], and colorectal cancers [13], and participates in tumor cell malignancy. HOXB5 knockdown increases breast cancer cell susceptibility to tamoxifen [26], whereas overexpression of its expression increases cisplatin resistance in non-small cell lung cancer [27]. The expression of HOXB5 was found to be up-regulated in ovarian cancer [28], which was consistent with the GEPIA database results, however, there was no research on its effects on the proliferation and metastasis of ovarian cancer cells. It so happens that our findings complement these.

According to research, blocking the NF-κB signaling pathway can improve ovarian cancer cell susceptibility to cisplatin [29]. As a consequence, it is speculated that CARD9 may be involved in cisplatin sensitivity of ovarian cancer via NF-κB. To further explore the probable regulatory mechanism of CARD9 in ovarian cancer, the effect of CARD9 on the activation of NF-κB signaling was evaluated. The results indicated that CARD9 knockdown hindered signal activation, whereas HOXB5 reversed its impact. This suggests that the NF-κB pathway is a downstream signal controlled by HOXB5/CARD9. It is worth mentioning that a study suggests that CARD9 causes the polarization of tumor-associated macrophages in colon cancer metastasis [30]. CARD9 is likely to play an essential role in regulating the tumor microenvironment, which will be the focus of our future research.

In conclusion, this study revealed that CARD9 is involved in ovarian cancer cell proliferation, migration, and cisplatin sensitivity via NF-κB signaling after transcriptional activation by HOXB5. The regulatory role of CARD9-dependent NF-κB signaling in ovarian cancer is disclosed, which lay the foundation for follow-up research.

## Methods and materials

### Sample collection

The Ethics Committee of the first Affiliated Hospital of Jinzhou Medical University approved the current study. All procedures were carried out in conformity with the 1964 Declaration of Helsinki. The subjects were informed of the project and signed an informed consent form. Ovarian cancer tissue and adjacent tissue were gathered from 15 patients. Inclusion criteria: age ≥ 18 years; no anti-tumor therapy prior to enrollment. Exclusion criteria: combined with tumors in other regions, systemic infections, serious disorders of vital organs, and autoimmune diseases. Total RNA and proteins were extracted from these samples followed by RT-qPCR and immunoblotting as detailed below.

### Cell culture

Ovarian epithelial cells IOSE-80 (EK-Bioscience, Shanghai), cancer cell lines OVCAR3 (ATCC), HEY (ATCC), and A2780 (ECACC) were cultured in RPMI-1640 (Gibco, ThermoFisher). Cancer cell line SKOV3 (ATCC) and cisplatin-resistant cell line SKOV3/DDP were cultured in McCoy’s 5a (Gibco). SKOV3/DDP was created utilizing the previously reported concentration-increasing approach [31] with cisplatin (C2210000, Merck). They were all supplemented with 10% fatal bovine serun and 1% penicillin-streptomycin (Gibco). Cells were grown at 37 °C with 5% CO2/95% air.

### Cell transfection

Specific CARD9 or HOXB5 shRNAs and scrambled shRNAs as negative controls (NC; GenePharma, Shanghai) were transfected into SKOV3 or SKOV3/DDP cells to achieve knockdown. To achieve overexpression, HOXB5 plasmids and empty plasmids (GenScript, Nanjing) as the NC were transfected into SKOV3 or SKOV3/DDP cells. The assay was operated following the product instruction manual of Lipofectamine 3000 transfection reagent (Invitrogen). The transfection mixture was replaced with fresh medium after 6 h of transfection, and efficacy was determined using RT-qPCR and immunoblotting as detailed below.

### RT-qPCR

TRIzol® reagent (Invitrogen) and LabScript RT kit (Biolab, Beijing) were used to extract total RNA from cells or tissues and generate cDNA. The QuantiTect SYBR Green PCR kit (Qiagen) was used for RT-qPCR. The relative mRNA levels were quantified using the ∆∆Ct method following normalization against GAPDH. Primer sequences are listed in Table 1.

**Table 1:** Primer sequences for RT-qPCR

Gene	Forward 5′-3′	Reverse 5′-3′
CARD9	CGGCGCCTCAAAGAGAGTT	CCCTCAGTGTCGGTGTTGTC
HOXB5	GTAAACTCCTTCTCGGGGCG	GAGAGCTGCCACTGCCATAA
GAPDH	GACTCATGACCACAGTCCATGC	AGAGGCAGGGATGATGTTCTG

### Immunoblotting

Protein was extracted from cells or tissues following RIPA lysis buffer (WellBio, Shanghai) treatment and quantified using Nano 300 protein detector (Allsheng, Hangzhou). The proteins were apart on the polyacrylamide gel through sodium dodecyl sulfate polyacrylamide gel electrophoresis and transferred to PVDF membranes (Roche). The membranes were incubated with skimmed milk for 1 h and then they were cut into strip-shaped blots. The blots were incubated with primary antibodies at 4 °C overnight followed by horse radish peroxidase-conjugated antibody for 1.5 h. Blots were visualized with an enhanced chemiluminescence solution (Sbjbio, Nanjing) and results were analyzed with ImageJ 1.52 software. Information for antibodies are listed in Table 2.

**Table 2:** Antibodies used for Immunoblotting

Antibody	Catalog number	Host	Dilution ratio	Company
CARD9	A305-878A-T	Rabbit	1:1000	Thermo Fisher
Ki67	Orb389335	Rabbit	1:500	Biorbyt
PCNA	Orb48485	Rabbit	1:1000	Biorbyt
MMP2	GTX59880	Rabbit	1:2000	GeneTex
MMP9	GTX100458	Rabbit	1:1000	GeneTex
CDK4	Orb48321	Rabbit	1:1000	Biorbyt
Cyclin D1	Orb33974	Rabbit	1:1000	Biorbyt
Bcl-2	AB112	Rabbit	1:1000	Beyotime
Bax	AF1270	Rabbit	1:2000	Beyotime
cleaved-caspase3	9661S	Rabbit	1:1000	CST
HOXB5	PA5-35898	Rabbit	1:500	Thermo Fisher
p-NF-κB p65	Ab76302	Rabbit	1:1000	Abcam
NF-κB p65	Ab16502	Rabbit	1:1000	Abcam
Lamin B1	Orb556089	Rabbit	1:1000	Biorbyt
GAPDH	GTX100118	Rabbit	1:50,000	GeneTex
anti-rabbit lgG (HRP)	A0208	goat	1:1000	Beyotime

### CCK-8

Cells in the logarithmic growth phase were trypsinized with 0.25% trypsin (Gibco) before being seeded into a 96-well plate for 24 h. CCK-8 reagent (Beyotime) was added to the wells and the cells were incubated at 37 °C for 1 h. Optical density was measured at 450 nm using a microplate reader (Perlong, Beijing).

### Colony formation

Cells were seeded into culture dishes at a density of 500 cells/dish. They were cultured for 2 weeks, with the medium changed every 3 days. Following that, the cells were rinsed twice with phosphate buffered saline (PBS), fixed with 4% paraformaldehyde (TCI, Shanghai), and stained with 0.5% crystal violet (Macklin, Shanghai). A colony was defined as a cluster of ≥50 cells.

### Cell migration

A wound healing assay was used to analyze migration. Cells were cultivated until they formed a confluent monolayer, then a wound in the center was created with a sterile pipette tip followed by another 24 h of culture. Results were observed under a microscope (magnification × 100, Olympus).

### Flow cytometry

The cell pellet was gently mixed with pre-chilled 70% ethanol and incubated overnight at 4 °C. Then cells were centrifuged and washed in ice-cold PBS. Propidium iodide stock solution and RNase A solution were added to the staining buffer provided with the kit (Yeasen, Shanghai) and mixed. The above-prepared staining solution was added to cells followed by incubation at 37 °C for 30 min in the dark. Flow cytometer (BD FACS Calibur, USA) and FlowJo7.6.1 software was used to determine the cell cycle distribution.

### Dual-luciferase reporter assay

CARD9 wild-type and mutant-type luciferase expression vectors containing HOXB5 binding sites were created and co-transfected into cells along with HOXB5 plasmids and empty plasmids. The Dual-luciferase Reporter Assay System (Promega) was used to measure luciferase activity, which was normalized to *Renilla* luciferase activity.

### ChIP

The association between CARD9 and HOXB5 was examined using a ChIP kit (Amylet, Wuhan, China). The cells were treated with 16% methanol, lysis buffer, and sonicated. The cells were then incubated overnight with an anti-HOXB5 or lgG antibody. Following incubation, protein A/G beads were used to harvest the protein-DNA complex and 5 mmol/l NaCl was used to retrieve the DNA. The level of CARD9 was determined using PCR.

### Bioinformatics and statistics analysis

The levels of CARD9 and HOXB5 in ovarian cancer, as well as their association with overall survival, were investigated using GEPIA. The correlation analysis between CARD9 and HOXB5 gene levels was based on the TCGA database. The binding location between CARD9 promotor and HOXB5 was obtained using the JASPAR database. GraphPad Prism 8.0 was utilized for statistical analysis. The data is presented as the mean ± SD (*n* ≥ 3). Statistical significance was determined via Student’s t-test and ANOVA with Tukey’s post-hoc test. *P* < 0.05 was considered to be statistically significant.

## Supplementary Information

**Additional file 1: Fig. S1.** Images of blots with molecular markers in Fig. 1. **Fig. S2.** Images of blots with molecular markers in Fig. 2. **Fig. S3.** Images of blots with molecular markers in Fig. 3. **Fig. S4.** Images of blots with molecular markers in Fig. 4. **Fig. S5.** Images of blots with molecular markers in Fig. 5. **Fig. S6.** Images of blots with molecular markers in Fig. 6.

## Data Availability

The dataset supporting the conclusions of this article is included within the article.
