# Supplementary material for: CARD9 contributes to ovarian cancer cell proliferation, cycle arrest, and cisplatin sensitivity
Source: BMC Mol Cell Biol. 2022 Nov 28;23:49. doi: 10.1186/s12860-022-00447-0 (PMC9703781; doi:10.1186/s12860-022-00447-0)
Supplement: Supplementary file 1 — Additional file 1: Fig. S1. Images of blots with molecular markers in Fig. 1. Fig. S2. Images of blots with molecular markers in Fig. 2. Fig. S3. Images of blots with molecular markers in Fig. 3. Fig. S4. Images of blots with molecular markers in Fig. 4. Fig. S5. Images of blots with molecular markers in Fig. 5. Fig. S6. Images of blots with molecular markers in Fig. 6. [file 12860_2022_447_MOESM1_ESM.pdf]

Supplementary file

CARD9 contributes to ovarian cancer cell proliferation, cycle arrest, and cisplatin sensitivity

By: Yanming Wang, Chao Wang, Yan Zhu

Figure S1 Images of blots with molecular markers in Figure 1

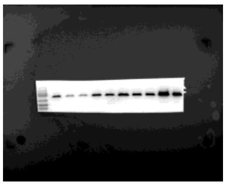
1D-CARD9
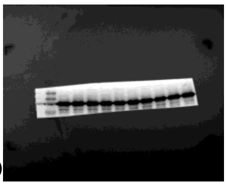
1D-GAPDH

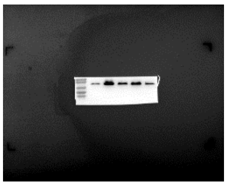
1F-CARD9
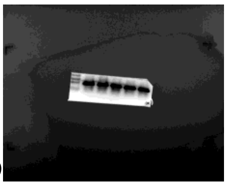
1F-GAPDH

Figure S2 Images of blots with molecular markers in Figure 2

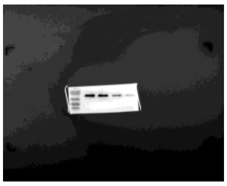
2B-CARD9
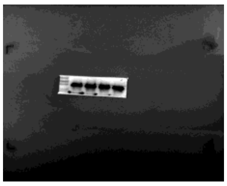
2B-GAPDH
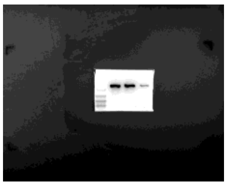
2F-Ki67

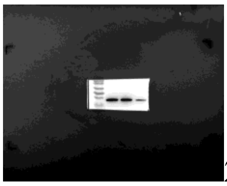
2F-PCNA
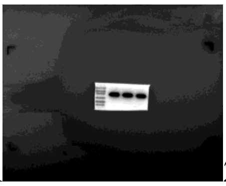
2F-LaminB1
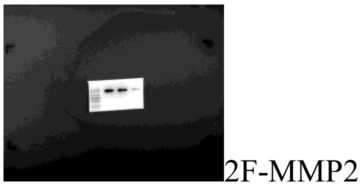
2F-MMP2

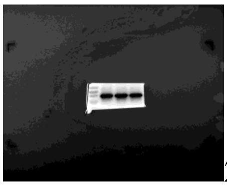
2F-GAPDH
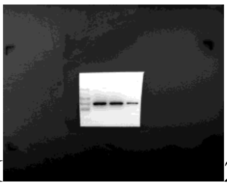
2F-MMP9
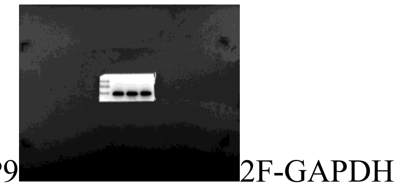
2F-GAPDH

Figure S3 Images of blots with molecular markers in Figure 3

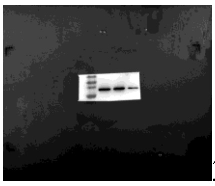
3B-CDK4

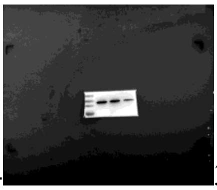
3B-CyclinD1

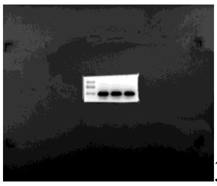
3B-GAPDH

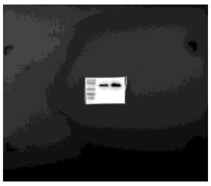
3D-CARD9

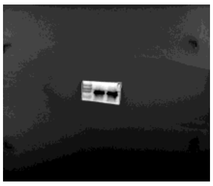
3D-GAPDH

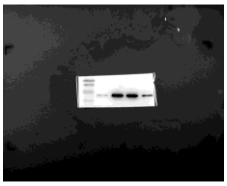
3H-Bcl2

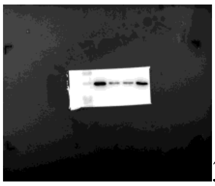
3H-Bax

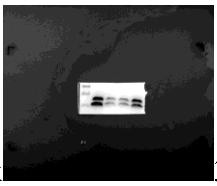
3H-cleaved cas3

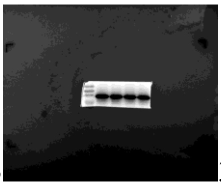
3H-GAPDH

Figure S4 Images of blots with molecular markers in Figure 4

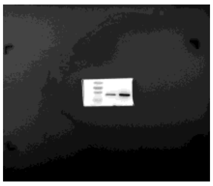
4C-HOXB5

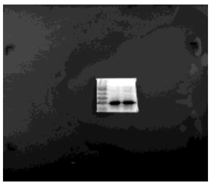
4C-GAPDH

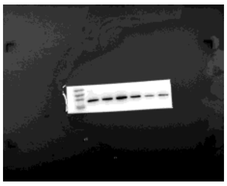
4F-HOXB5

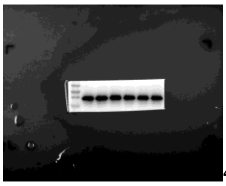
4F-GAPDH

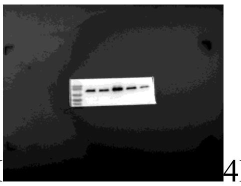
4H-CARD9

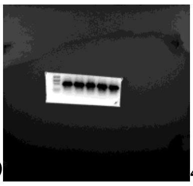
4H-GAPDH

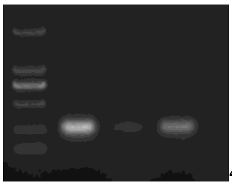
4K

Figure S5 Images of blots with molecular markers in Figure 5

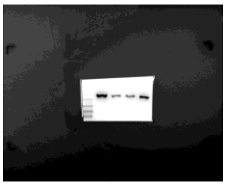
5D-Ki67

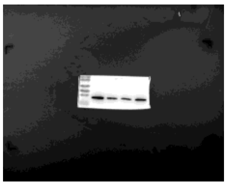
5D-PCNA

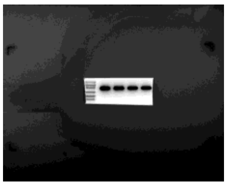
5D-LaminB1

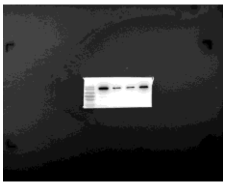
5D-MMP2

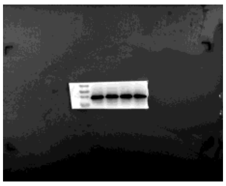
5D-GAPDH

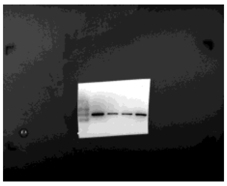
5D-MMP9

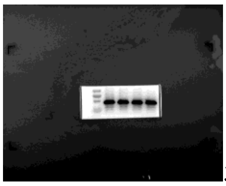
5D-GAPDH

Figure S6 Images of blots with molecular markers in Figure 6

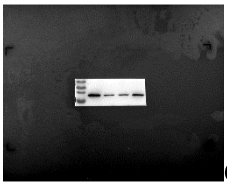
6B-CDK4

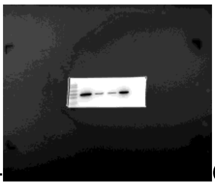
6B-CyclinD1

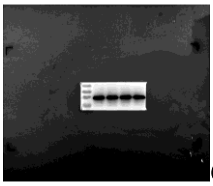
6B-GAPDH

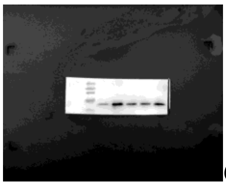
6E-Bcl2

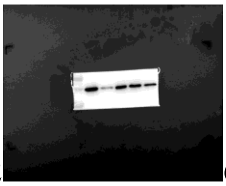
6E-Bax

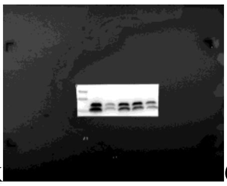
6E-cleaved cas3

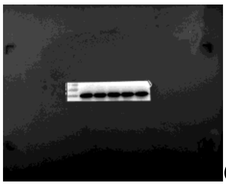
6E-GAPDH

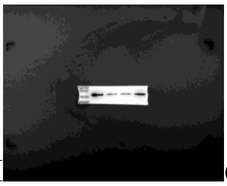
6F-p-NFκB p65

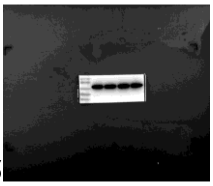
6F-NFκB p65

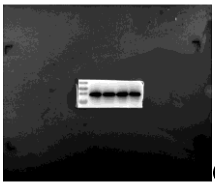
6F-GAPDH
